# How Political Science Can Contribute to Public Health: A Response to Gagnon and Colleagues

**DOI:** 10.15171/ijhpm.2017.103

**Published:** 2017-09-06

**Authors:** Anita Kothari, Ruta Valaitis, Vera Etches, Marc Lefebvre, Cal Martell, Sinéad McElhone, Ruth Sanderson, Louise Simmons

**Affiliations:** ^1^School of Health Studies, Faculty of Health Sciences, Western University, London, ON, Canada.; ^2^School of Nursing, McMaster University, Hamilton, ON, Canada.; ^3^Ottawa Public Health, Ottawa, ON, Canada.; ^4^Sudbury & District Health Unit, Sudbury, ON, Canada.; ^5^Champlain LHIN, Ottawa, ON, Canada.; ^6^Public Health Department-Niagara Region, Niagara, ON, Canada.; ^7^Oxford County Public Health, Woodstock, ON, Canada.; ^8^Eastern Ontario Health Unit, Cornwall, ON, Canada.

## Dear Editor,

As public health scholars and practitioners, we read the perspective offered in “*Why and How Political Science Can Contribute to Public Health? Proposals for Collaborative Research Avenues”* by Gagnon et al^1^ with great interest. The authors start by noting the divide between political science and public health, but point out that both fields are interested in activities that support “the public good” (ie, health). The convergence lies in the common interests of institutions, public administration, governance and policy analysis. The main purpose of the article is to suggest how political science can contribute to public health science, policy and practice. Research about healthy public policies tends to ignore the political. To move forward, the authors propose areas for future collaborative research.

Our intent is to clarify and build on the discussion drawing from our years of experience in public health research and practice. We are a research team made up of practitioners from five health units, academics from two universities, a medical officer of health and a director from a local health planning agency. Many of us have sat on key policy advisory committees at provincial and national levels. Our project is funded by a unique grant program that supports applied, collaborative research partnerships (https://www.publichealthontario.ca/ldcp). This commentary caught our eye because our project was rapidly developed and funded by Public Health Ontario, an arm’s length operational service agency of the provincial government in response to a new law in Ontario, called the *Patients First Act*. The Act requires public health units to work with local health planning agencies and use a population health approach to plan health services for a community. We are exploring values, goals, definitions, processes, structures and use of population health information to determine the key elements of successful public health unit and regional health planning agency collaborations. We are also identifying relevant population and health system types and sources of information to inform indicators that could strengthen these collaborations. Thus, our work sits in the research domain of public health systems and services, and we are generating research findings to contribute to how the policy will be implemented in the province. This is work with immediate relevance.

First, we offer a minor but important clarification. On page 2, the article states that, “In all countries, protection of the population from health risks is the traditional and main mission of public health.” In addition to disease and injury risk protection, the core functions of public health include population health assessment, surveillance, health promotion and health protection, with a mandate to decrease health inequities through a focus on the social determinants of health.^2^ This is important because the aim of decreasing health inequities speaks to a value that accompanies public health research and practice: this drive for social justice needs to be acknowledged by those who work with public health researchers and practitioners. An implication of this value is that we engage the public health community to develop research studies and practice (strategies, services and policies) that are relevant and feasible in the lives of community members or equity-seeking populations. In our project this has been accomplished by on-going engagement with various decision-making bodies,* even before the work was funded*, to raise awareness about the project, and to ensure the research question and approach would meet the needs of various actors – eg, frontline staff, senior decision-makers in and outside of local and provincial governments – involved in the implementation of the policy.

The second point draws from Van de Ven and Johnson^3^ to suggest that the knowledge generated by research is different than that produced by practice. That is, researchers and practitioners are part of distinct epistemological communities. Practitioner knowledge, about understanding in action, is a distinct form of knowledge in its own right while research knowledge relates to moving from a specific to a general understanding. Van de Ven and Johnson see, as do we, these knowledge sources as equal yet complementary, each with its own standards of relevance and rigour. We raise this point because most of the “divergent views” between political science and public health research presented by Gagnon et al are explained by this perspective. For example, “that political scientists are interested in analyzing the roles and responsibilities of actors in public institutions and governance versus the public health interest in using the roles and responsibilities of public health actors to advocate for social and policy change” – these are essentially the differences between research and practice. We would reframe this divergence as the need for researchers from both political science and public health/epidemiology/health promotion to recognize and promote the need for pluralistic and collaborative approaches to knowledge production. An example of this approach is when practitioners, researchers and government officials came together to develop an agenda for public health systems research.^4^ Another example is public health ethics, a new area of scholarship which might be showcased as an exemplar case of strong interdisciplinary and practice participation.^5,6^ As a final example, our own research team is made up of practitioners and academics so that we can understand the public health problem in context. We suggest that political science might intersect with public health in stronger ways if political science scholars embrace the idea that much of public health research is theoretically-informed, applied research done with a political lens, while public health practice is about solving a specific problem in a specific context and is often driven by policy. Both approaches are essential.

We conclude with a challenge. Gagnon et al present their perspective on how public health can be informed by political science by: (*a*) an appreciation of the political context of our societies, (*b*) exploring the functions of public health at various levels of the system, (*c*) evaluating public health policies, and (*d*) conducting comparative analyses of regions. We suggest that these topics can remain in the scholarly domain if the goal is to “anchor the analysis of public health policies in political science approaches and tools.” But if political science research wants to claim impact through improvements in the health of the population and decreasing health inequities, the field needs to determine how these high level research objectives can be relevant for local jurisdictions. We have offered some suggestions about how this can be accomplished.

## Acknowledgement

The “*Public Health Units and LHINs Working Together for Population Health*” Project Team would like to thank PHO for its support of this project. The team gratefully acknowledges funding received from PHO through the Locally Driven Collaborative Projects program. The views expressed in this publication are the views of the project team, and do not necessarily reflect those of PHO.

## Ethical issues

Not applicable.

## Competing interests

Authors declare that they have no competing interests.

## Authors’ contributions

AK conceived the idea and wrote the first draft of the letter, RV contributed important edits and VE, ML, CM, SM, RS, and LS provided feedback on drafts. All authors reviewed the final version.

## Authors’ affiliations

^1^School of Health Studies, Faculty of Health Sciences, Western University, London, ON, Canada. ^2^School of Nursing, McMaster University, Hamilton, ON, Canada. ^3^Ottawa Public Health, Ottawa, ON, Canada. ^4^Sudbury & District Health Unit, Sudbury, ON, Canada. ^5^Champlain LHIN, Ottawa, ON, Canada. ^6^Public Health Department-Niagara Region, Niagara, ON, Canada. ^7^Oxford County Public Health, Woodstock, ON, Canada. ^8^Eastern Ontario Health Unit, Cornwall, ON, Canada.
